# Exploring the Impact of COVID-19 on the Sustainability of Health Critical Care Systems in South America

**DOI:** 10.34172/ijhpm.2020.116

**Published:** 2020-07-05

**Authors:** Fernando Almeida

**Affiliations:** ^1^University of Porto, Porto, Portugal.; ^2^INESC TEC - Institute for Systems and Computer Engineering, Technology and Science, Porto, Portugal.; ^3^ISPGAYA - Polytechnic Higher Institute of Gaya, Vila Nova de Gaia, Portugal.

**Keywords:** Coronavirus, Latin Countries, Pandemic, Intensive Care

## Abstract

The coronavirus disease 2019 (COVID-19) pandemic has created strong pressure on national health critical care systems. After its initial impact in Asia, the highest case growth is now in the Americas. The South American countries face a strong challenge due to the vulnerabilities of their health systems and the fragile socio-economic conditions of their population. This perspective looks at the impact of COVID-19 in South America and argues that the health critical care systems of these countries are particularly vulnerable due to the underestimation of the number of cases currently confirmed and the strong need for treatment of these patients in intensive care units (ICUs). In particular, Bolivia will need to increase the number of ICU beds 60-fold while Brazil will need to grow 12-fold to meet the growth rates of COVID-19 by the end of July 2020. In this sense, it is argued that national and transnational measures should be taken urgently to face this challenge. Furthermore, it is necessary to perform tests to detect COVID-19 cases earlier to alleviate the need for internment in ICUs.

## Introduction

The coronavirus disease 2019 (COVID-19) pandemic, caused by the severe acute respiratory syndrome coronavirus 2 (SARS-CoV-2) virus, has been impacting on a global scale. The repercussions of this pandemic are multifaceted and have challenged the sustainability of national health systems.^1,2^ The issues of health system sustainability are related to the quality of the system and its ability to meet demand without compromising unreasonable cost or debt growth.^3^

Although COVID-19 is not a virus with a high death rate, it exhibits a high level of contagion.^4^ The greatest part of fatalities of COVID-19 appears in elderly people or those with weak immunity. However, the high spread of the new coronavirus can have a drastic impact on health critical care systems. Hospitals may become overcrowded and unable to cope with strong demand in a short period of time. The poorest countries, with structural deficiencies and weak public health systems, will be the most affected.^5,6^ Currently the epicenter of COVID-19 is in the American continent, and due to fragile socio-economic conditions and high levels of poverty, a strong impact of COVID-19 is expected in South America.^7^ Furthermore, the tests are insufficient and are often performed only on patients in advanced stages of the disease. To meet this challenge, some countries like Chile consider it important to change the testing paradigm to enable them to be performed in primary healthcare.^8^ In this way, there is greater proximity between hospital units and the population, which necessarily facilitates access to testing for the most vulnerable populations.

South America is a continent that has undergone profound changes in recent years. Several South American countries, such as Brazil, Argentina, Chile, or Colombia, have taken advantage of national and regional contexts motivated by the end of military dictatorships and socio-economic crises, characterized by high levels of social injustice to drive health system reforms. The implementation of various public policies has extended the coverage of health services to the most vulnerable populations, improving access to health.^9^ Despite advances, health systems in South America retain structural characteristics of fragmentation in the organization and delivery of services. The segmentation of financing has led to inequities and inefficiencies in these health systems.^9^ The coronavirus pandemic in South America challenges health systems and the financial capacity of their governments and raises fears about the spread of COVID-19, especially among the poorest and most vulnerable population.

 This paper aims to respond to the gap in the analysis of the incidence of COVID-19 in six South American countries (ie, Argentina, Bolivia, Brazil, Chile, Colombia, Peru). These countries have felt very heterogeneous impacts of COVID-19 and have responded to this challenge in a very asymmetric way. Accordingly, it is pertinent to explore the pressure on the health critical care system that will be placed on these countries considering the number of current cases and a projection of disease evolution. This study is pertinent and timely considering the current pandemic scenario and the alarming growth in the number of cases in South America.

## Methods

 This paper intends to analyze the proliferation of COVID-19 pandemic in South American countries. Furthermore, it aims to explore the capacity of these health critical care systems to cope with the current number of cases of COVID-19 and the expectations of growth in the coming months.

To explore the impact of the propagation of the COVID-19 pandemic and to assess its impacts on national healthcare systems in South American countries, the database provided by the Institute for Health Metrics and Evaluation (IHME) was employed. The IHME is a leading health research institute founded by the Bill and Melinda Gates Foundation and provides global health statistics and health-related sustainable development goals indicators that measure the level of development of health systems in various countries.^10^

The data provided by the IHME do not include all countries in South America. Therefore, the sample considered for this study included only data from 6 countries. For each country, the number of confirmed cases and deaths was recorded. The estimated number of infections was also recorded considering the number of tests performed in each country. Estimated cases include both untested and asymptomatic individuals. Projections of the number of infected were carried out by IHME. After that, the case fatality rate (CFR) and the infection fatality rate (IFR) were subsequently calculated. The CFR considers the number of deaths taking into account the total number of individuals diagnosed with COVID-19, while in the IFR all potentially infected individuals are considered.^11^ Finally, the number of available beds and the intensive care unit (ICU) available beds dedicated to COVID-19 were considered when identifying available hospital resources. This data is mapped in Table 1. For each indicator, the absolute value and the percentage value (%) per 100k are given.

**Table 1:** Analysis of COVID-19 in South America on May 31, 2020

**Country**	**Deaths ** **No. (%)**	**Confirmed Cases ** **No. (%)**	**Estimated Cases ** **No. (%)**	**CFR **	**IFR **	**Beds Available** **No. (%)**	**ICU Available Beds (%)**
Argentina	543 (1.20)	16851 (37.31)	58039 (128.50)	0.0322	0.0094	52482 (116.33)	4288 (9.50)
Bolivia	337 (2.81)	9592 (82.25)	263682 (2261.05)	0.0351	0.0013	7117 (59.25)	54 (0.45)
Brazil	28881 (13.33)	514849 (242.33)	8218441 (3868.24)	0.0561	0.0035	83774 (38.67)	4060 (1.87)
Chile	1072 (5.89)	99688 (521.78)	191781 (1003.81)	0.0108	0.0056	9578 (52.63)	396 (2.18)
Colombia	924 (1.93)	29383 (57.79)	973698 (1915.00)	0.0314	0.0009	22767 (47.65)	1757 (3.68)
Peru	4506 (13.68)	164476 (499.32)	2695066 (8181.80)	0.0274	0.0017	17265 (50.79)	88 (0.26)

Abbreviations: COVID-19, coronavirus disease 2019; CFR, case fatality rate; IFR, infection fatality rate; ICU, intensive care unit.

The impact of COVID-19 in South America was quite diverse. The highest number of deaths was recorded in Brazil, but the mortality rate is slightly higher in Peru. In contrast, Argentina and Colombia have the lowest mortality rates. The highest number of confirmed cases is found in Chile, which indicates a higher percentage of tests performed in the population.^12^ Although the number of confirmed cases in Bolivia is lower than in Chile, the estimated number of cases in percentage is twice that of Chile. The CFR in Brazil is approximately 5 times higher than in Chile, although the IFR is about 40% lower in Brazil. Finally, the number of beds available in healthcare is also very heterogeneous. The largest number of available beds is in Argentina, both outpatient and ICU; while Peru and Bolivia are two countries with very few ICU beds.

## Healthcare System Capacity

Data on the number of beds available in ambulatory and ICU already give an idea of the high pressure that some countries in South America (eg, Bolivia, Peru) may feel on their health systems. However, to understand in-depth the magnitude of the problem, Table 2 presents a projection on the number of beds needed in each country. Two scenarios for different periods were considered: May 31, 2020 and July 31, 2020. The second scenario considers the projection of the evolution of the disease in each country according to the IHME model.

**Table 2:** Projection of the Evolution of the COVID-19 and the Response Capacity of the National Health Systems

**Country**	**Scenario I**		**Scenario II**	
	**All Beds Needed (%)**	**ICU Beds Needed (%)**	**All Beds Needed (%)**	**ICU Beds Needed (%)**
Argentina	434 (0.96)	122 (0.27)	3484 (7.72)	914 (2.03)
Bolivia	757 (6.30)	172 (1.43)	14093 (117.33)	3251 (27.07)
Brazil	39414 (18.19)	10381 (4.79)	194673 (89.85)	49034 (22.63)
Chile	2076 (11.41)	568 (3.12)	10235 (56.24)	2696 (14.81)
Colombia	1460 (3.06)	373 (0.78)	17508 (36.65)	4546 (9.52)
Peru	7694 (22.63)	2018 (5.94)	n/a	n/a

Abbreviations: COVID-19, coronavirus disease 2019; ICU, intensive care unit.

The scenario I demonstrates the current pressure on health systems in these countries. In countries such as Bolivia, Brazil, Chile, and Peru, the number of ICU beds available was lower than the number of beds needed given the severity of symptoms experienced by patients. These results confirm previous studies in Brazil, where it is estimated that ICUs would be particularly affected due to the insufficient number of beds available.^13^ This situation is already visible in states like Amazonas and São Paulo that feel great pressure from serious cases of COVID-19 that need hospital internment.^14^ In this sense, the data from each country only provide a general perception of the incidence rate, but local challenges emerge that need urgent responses, particularly in poorer states where financial resources are scarcer.

 In the scenario II, Argentina is the only country expected to have sufficient capacity of ICU beds to meet the demand of the population. In Argentina, it is estimated that the existing capacity is 4 times greater than the need. On the contrary, in Bolivia, the necessary capacity of ICU beds would have to be 60 times larger to meet the estimated needs and, in Brazil, a capacity 12 times larger would be needed.

## Conclusion

 South America is currently an epicenter of the COVID-19 pandemic. The impact of the pandemic in these countries is quite diverse, having affected mainly countries like Brazil and Peru. The CFR and IFR are also indicators where the performance of countries is quite heterogeneous. In several countries like Bolivia and Colombia, there is a very significant difference between the number of registered and estimated cases. The number of COVID-19 tests performed in each country is an important element to decrease the asymmetry between these two indicators and to allow treating patients still at an early stage of the disease and avoiding the need for more evasive interventions in ICUs. Furthermore, the pressure on national health systems in South American countries is already quite high, especially among ICUs. End of July data indicates that several countries (eg, Bolivia, Brazil, Chile, and Peru) do not have sufficient capacity to receive all the necessary internments. This situation is particularly critical in Bolivia or Peru given the very low number of ICU available beds to treat COVID-19 patients. However, this situation is likely to worsen considering the IHME’s forecasts. In Brazil, it is estimated that about 50000 beds are needed in ICUs, while only about 4000 beds are available throughout the country.

 This study essentially provides an overview of the impact of COVID-19 in South America and analyzes the current and estimated pressure on national health critical care systems. With this, it is intended to foster the emergence of national and transnational public policies that help mitigate the consequences of the pandemic on the healthcare systems of these countries.

## Ethical issues

 Not applicable.

## Competing interests

 Author declares that he has no competing interests.

## Author’s contribution

 FA is the single author of the paper.
